# The use of a short course of Ulipristal Acetate for acute abnormal uterine bleeding in women without uterine fibroids

**DOI:** 10.52054/FVVO.15.2.078

**Published:** 2023-06-30

**Authors:** I Lambrecht, T Van den Bosch

**Affiliations:** Department of Obstetrics & Gynaecology, UZ Leuven, Herestraat 49, 3000 Leuven, Belgium; Department of Development and Regeneration, KU Leuven, Herestraat 49, 3000 Leuven Belgium

**Keywords:** Ulipristal Acetate, pharmacokinetics, fibroids, abnormal uterine bleeding

## Abstract

**Background:**

Ulipristal Acetate (UPA) is a synthetic selective progesterone receptor modulator. It is used as emergency contraception and to reduce pain and blood loss in women of reproductive age with uterine fibroids. The first mechanism of action is myometrial apoptosis, the second is on the hypo-thalamic-pituitary-ovarian axis and the third action, is an anti-proliferative effect on the endometrium. Mainly based on the latter two, UPA is increasingly used off-label in women with abnormal uterine bleeding (AUB) without fibroids.

**Objectives:**

The aim of this paper is to find evidence for a short course of UPA to treat acute AUB without fibroids, performing a systematic review as well as scrutinising literature data on the pharmacokinetics and on short term bleeding control in women with fibroids.

**Materials and Methods:**

A systematic electronic literature review was performed in February 2022. Inclusion criteria were UPA administered to women without myomas in a setting of acute uterine bleeding. Further criteria included papers describing early bleeding control using UPA, deemed independent of the presence of fibroids, with specific attention to the median time to amenorrhoea.

**Main outcome measures:**

The main outcome measured was the bleeding control within 10 days.

**Results:**

One case report was identified. The data on symptomatic women with fibroids using 5 mg or 10 mg daily revealed bleeding control was reported within 10 days in 81% and 89% respectively, with amenorrhoea in 57% and in 78% respectively.

**Conclusion:**

A short-term administration may prove effective in abnormal uterine bleeding irrespective of the presence of uterine fibroids. However, more randomised controlled trials are needed and should be performed before implementation in general clinical practice.

**What is new?:**

A short course of Ulipristal acetate as promising treatment for acute uterine bleeding without fibroids.

## Introduction

Abnormal uterine bleeding (AUB) occurs in 8–27% of women of childbearing age. The most common presentation of abnormal bleeding is heavy menstrual bleeding, i.e., excessive menstrual blood loss interfering with the woman’s physical and social quality of life (Estadella et al., 2018).

Ulipristal Acetate (UPA) is a synthetic Selective Progesterone Receptor Modulator (SPRM) with progesterone agonist actions but predominantly antagonist effects because of a tissue-specific interaction with co-activators and co-repressors (Powell and Dutta, 2016).

UPA was first studied in the 1990’s in the context of an ‘anti-fertility’ drug. UPA was initially labelled as an ‘anti-progestin’, later it became classified as a SPRM. UPA is a steroidal SPRM with the structure of a 19 nor-progesterone derivative (Critchley and Chodankar, 2020).

After oral intake, it is quickly transformed into its active metabolite (N-demethylated) with a median time to Cmax (maximum of plasma concentration) of one hour. Clearance terminal elimination half-life is 32 ± 6 hours (Croxtall, 2012).

UPA is known to reduce pain and blood loss in women of reproductive age with uterine fibroids based on 3 levels of action. The first affect is the downregulation of the angiogenic growth factors resulting in apoptosis of the myometrial cells and inhibition of collagen synthesis, leading to shrinking of the myoma volume (Estadella et al., 2018). The second action is on the hypothalamic-pituitary- ovarian axis, creating low levels of LH and FSH, with a mid-follicular level of oestradiol (Estadella et al., 2018). The third target is the endometrium, altering the expression of the progesterone and androgen receptors in an anti-proliferative way (Estadella et al., 2018).

The last two levels of action indicate a favourable effect on heavy endometrial bleeding which may also be expected in the absence of myomas. Therefore, UPA is increasingly used off-label in women with acute heavy menstrual bleeding.

Known side effects of UPA are osteopenia or even osteoporosis with long-term use (Powell and Dutta, 2016). Additionally, some cases of severe hepatotoxicity have been described during chronic use of UPA. Therefore, the European Medicines Agency (EMA) decided in 2020 to discourage the long-term use of UPA. UPA is still on the market as emergency contraception in single 30 mg dose to be taken within 120 hours after unprotected intercourse (Critchley and Chodankar, 2020). The most common (1 in 10 patients experience) side effects after a single 30 mg dose are usually mild and include nausea, stomach pain, vomiting, menstruation pain, sensitive breasts, headache, dizziness, mood swings, muscle pain and fatigue (Croxtall, 2012).

The aim of this review is a systematic search for literature about the use of UPA for acute uterine bleeding for women of reproductive age without fibroids. Secondly, the data about its use in women with fibroids is searched to estimate its rapidity of bleeding control to predict the effect in a non- fibroid setting.

## Methods

A systematic electronic literature search with a KU Leuven login started in February 2022 and ended in April 2022. One person (IL) searched and selected the articles. Inclusion criteria were met when Ulipristal Acetate was administered to women without myomas in a setting of acute uterine bleeding.

Exclusion criteria for data included women with myomas or data about UPA in other settings such as its use as contraception or combined with long- acting reversible contraception (LARC).

No language restrictions were applied. All participants were pre-menopausal women of 18 years and older. All types of articles were included, randomised controlled trials, systematic reviews, case reports and cohort studies.

**Figure 1:**
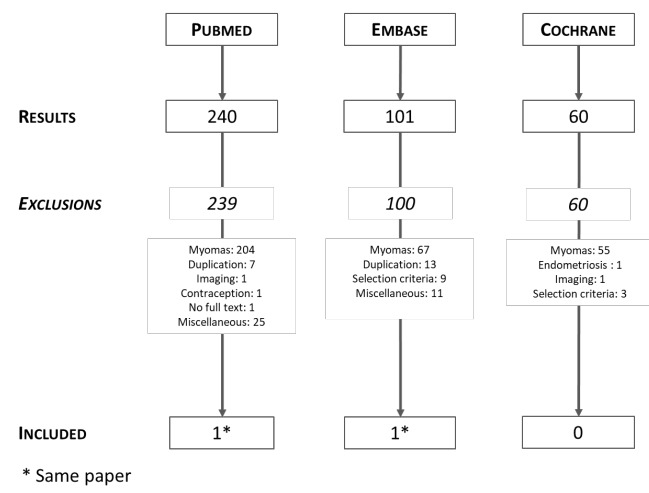
Flow diagram of the literature search. * Estadella et al., 2018.

The search was performed through MEDLINE via PubMed, Embase and the Cochrane Library (Figure 1). The PubMed search, with searching terms ‘ulipristal acetate’ and ‘bleeding’; ‘ulipristal acetate’ and ‘adenomyosis’; ‘ulipristal acetate’ and ‘heavy menstrual bleeding’; ‘ulipristal acetate’ and ‘bleeding control’ resulted in 127, 11, 57, and 45 papers respectively. Embase was searched for ‘ulipristal acetate’ and ‘bleeding’; ‘ulipristal acetate’ and ‘adenomyosis’; ‘ulipristal acetate’ and ‘without fibroids’; ‘ulipristal acetate’ and ‘no fibroids’, this resulted in 75, 25, 1 and 0 articles respectively. The Cochrane Library was used to search more articles, with searching terms ‘ulipristal acetate’ and bleeding’; ‘ulipristal acetate’ and ‘without fibroids’; ‘ulipristal acetate’ and ‘no fibroids’, yielding 31, 27 and 2 results respectively.

In a second stage to prepare for this paper, we scrutinised literature data on the pharmacokinetics of UPA. The results of the systematic search, excluded because the study population consisted of patients with fibroids, have been used in this part of the paper for the analysis of the mechanism, therapeutic response and expected effect of Ulipristal acetate deemed independent of the presence of fibroids.

## Results

The systematic literature review yielded one suitable article. Namely a case report by Estadella et al. (2018) describing a patient at the Hospital de la Santa Creu i Sant Pau in Madrid.

It reports the use of Ulipristal acetate in a 34-year- old woman with acute heavy menstrual bleeding. The patient has type-1 diabetes mellitus and multiple other co-morbidities. She presented with acute heavy bleeding and severe anaemia (haemoglobin 4.2 g/dL). Endometrial sampling did not show any endometrial pathology. Initially a blood transfusion was administered, and a Levonorgestrel Intra-Uterine Device (LNG-IUD) had been inserted. Forty- eight hours later, the heavy bleeding persisted, and the LNG-IUD was spontaneously expulsed. A new LNG-IUD was placed. Two days later, the heavy bleeding continued, the haemoglobin had dropped again (5.9 g/dL) and ultrasound examination confirmed the LNG-IUD had been expulsed for a second time. Due to the multiple comorbidities, combined hormonal contraceptives and antifibrinolytics were contraindicated, so 5 mg UPA was daily administered off-label for 12 weeks. Six days after the start of UPA, bleeding stopped. Follow-up was performed at one week, one month and two months post treatment. There was no relapse. After completion of treatment, a third LNG-IUD was inserted which resulted in adequate bleeding control in the following 6 months (Estadella et al., 2018).

### Myomas

To investigate the therapeutic potential of Ulipristal acetate in acute bleeding without myomas, we searched data for ‘time to bleeding control’ in myoma cases. Six papers were found describing the interval of bleeding cessation and/ or control.

One of the papers was an observational trial (Chen et al., 2017). 60 women with a low-GRADE quality level, the other 5 are publications about the PEARL-studies with a high-GRADE quality level. Donnez et al. (2012a; 2012b; 2014; 2015; 2016) published 4 randomised controlled trials on the use of UPA in women with fibroids and excessive uterine bleeding: the PEARL I, II, III and IV studies and the PEARL IV extension with 242,307,209 and 451 included women respectively of which 1 is not blinded (i.e. PEARL III) (Donnez et al., 2012a; Donnez et al., 2012b; Donnez et al., 2014; Donnez et al., 2015; Donnez et al., 2016). These publications, published in the New England Journal of Medicine and Fertility and Sterility, are high quality randomised controlled trials and contain the most important research data of UPA for fibroids. Part of the data presented below is deduced from those papers’ figures. The remaining studies focused on amenorrhoea and quality of life after 12 to 24 weeks UPA administration but provided no details on early bleeding control.

The PEARL I study is a randomised, double- blind, placebo-controlled phase 3 trial comparing the daily administration of 10 mg Ulipristal acetate (n=98) to 5 mg (n=96) and to placebo (n=48). Approximately 50% of the patients in the 5-mg group and 70% of those in the 10-mg group became amenorrhoeic within the first 10 days. The 10 mg dose induced amenorrhoea slightly faster compared to the 5 mg dose. Bleeding control, defined as subsequent pictorial blood loss assessment chart (PBAC) score < 75, was achieved within 7 days in 76% on 5mg, and 83% on 10 mg vs 6% for placebo (Whitaker et al., 2014).

**Figure 2:**
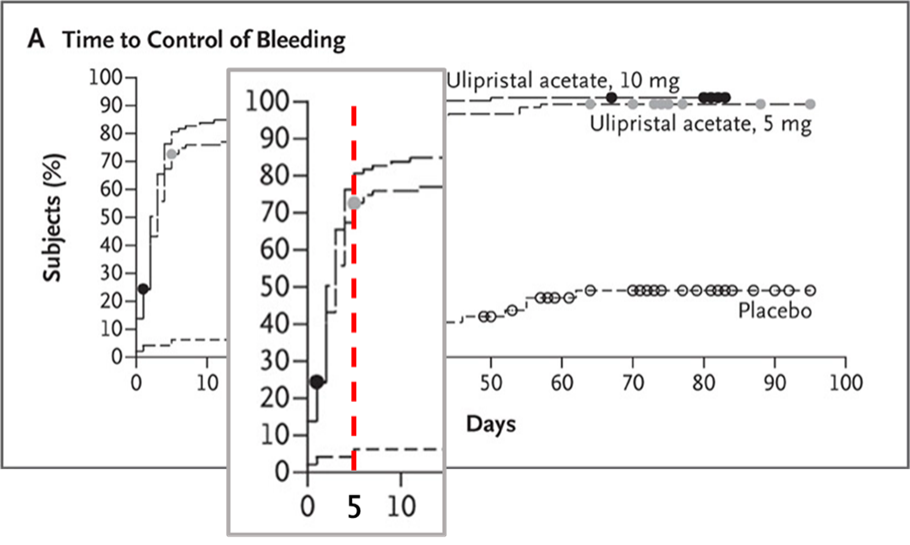
(Adapted from Donnez et al. 2012a - Pearl I) Bleeding control, defined as subsequent PBAC score <75, focussing on the first 10 days (vertical red dotted line indicating day 10).

As illustrated in Figure 2, by day 10, bleeding control was reported in 76% and 84% for 5 and 10 mg respectively (Figure 2). The steepness of the Kaplan-Meier curve graphically reflects the rapidity of bleeding control. However, the latter was not defined as primary nor secondary outcome in the PEARL I study (Donnez et al., 2012a).

**Figure 3:**
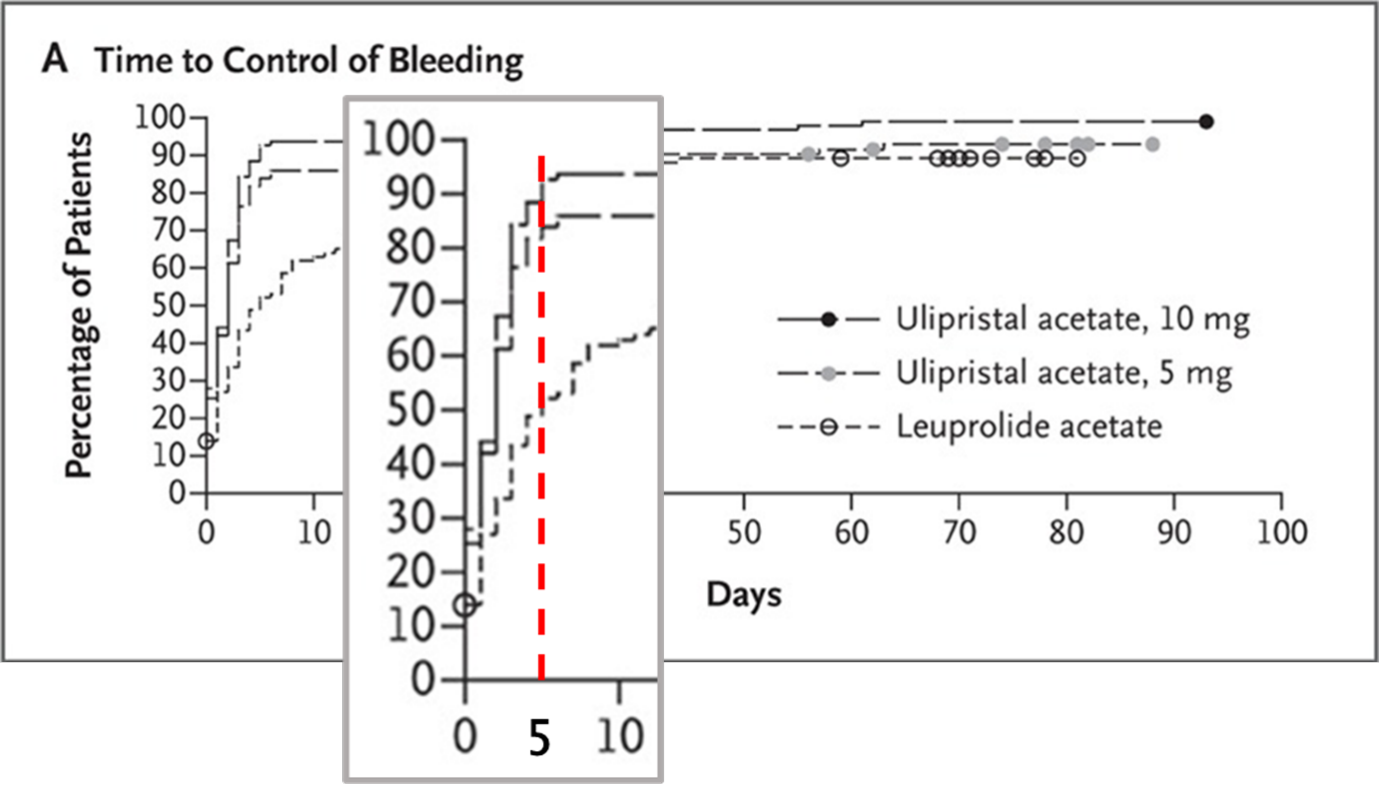
(Adapted from Donnez et al., 2012b - Pearl II): Time to bleeding control (PBAC-sore < 75), focussing on the first 10 days (vertical red dotted line indicating day 10).

The PEARL II study is a double-blind, non- inferiority trial in 307 patients with fibroids and excessive uterine bleeding (PBAC >100) receiving 3 months daily 5 mg UPA (n=98), 10 mg UPA (n=104) or monthly injection of Leuprolide, a GnRH-analogue. Median time to reach amenorrhoea was 7 days for patients receiving 5 mg UPA, 5 days for 10 mg and 21 days for those receiving Leuprolide acetate. By day 10, approximately 80% of the 10 mg-group and 57% of the 5 mg-group versus 30% with Leuprolide had amenorrhoea. The authors mention that the cause of the rapid effect on bleeding symptoms may be related to its effect on the endometrium (Donnez et al., 2012b). Bleeding control (PBAC- sore < 75) was reached within the first 10 days, in 86% of the 5 mg-group and 93% of the 10 mg-group. The highest dose had a slightly more rapid effect (Figure 3). Compared to Leuprolide, UPA induces amenorrhoea more rapidly and is associated with less adverse effects (Donnez et al., 2012b).

In the PEARL III Study, 209 women with uterine fibroids and heavy menstrual bleeding were included. The administration of repeated intermittent open-label 3-months course of 10 mg daily UPA, was followed by a randomised double-blinded and placebo- controlled 10-day administration of progestin (i.e., Norethisterone Acetate, NETA) after the end of each UPA treatment course. The aim of the additional NETA was to investigate its effect on progesterone receptor modulator associated endometrial changes (PAEC). The median time to amenorrhoea from treatment start was 3.5 days (interquartile range [IQR], 2–6 days). The median time to amenorrhoea after the start of each course was 2, 3, and 3 days for the 2nd, 3rd, and 4th course respectively (Donnez et al., 2014). By day 10, amenorrhoea was present in about 65% in the first course and approximately 86%, 80%, 86% in the 2nd, 3rd and 4th course respectively.

The PEARL IV study is a randomised, double-blind study on the efficacy and safety of two 12-week courses of UPA (5 or 10 mg) in 451 patients with heavy menstrual bleeding. The administration started in the first 4 days of menstruation. The median time to amenorrhoea after start of treatment was ≤ 6 days for each treatment group: 5 days (2-9) in the 5 mg-group versus 4 days (2-7) in the 10 mg-group during the first course, and 5 days (4-9) versus 6 days (4-8) days respectively during the second course. The differences between the two dosages did not reach significance (Donnez et al., 2015).

The PEARL IV extension publication includes the results after an additional 3rd and 4th 12-week course of UPA. The median times to amenorrhoea was 6 and 5 days for treatment courses 3 and 4 respectively both for the 5 mg and 10 mg dosages (Donnez et al., 2016).

In an observational study by Chen et al., including 60 women, aged 25-58 year with uterine fibroids, 64% became amenorrhoeic within the first 10 days of 5 mg UPA daily. One patient stopped treatment due to aggravation of bleeding (Chen et al., 2017).

### Adenomyosis

In women with adenomyosis, short term effects of UPA have not been reported yet.

Conway et al. 2019 reported on 6 premenopausal women with adenomyosis, in whom adenomyosis had erroneously be suspected, taking 5 mg UPA a day for 3 months. All 6 patients became amenorrhoeic. However, the prolonged administration of UPA for 3 months caused an increase in pelvic pain. On ultrasound, the adenomyosis features did increase over the 3 months period (Conway et al., 2019).

In a multicentre double blinded randomised placebo-controlled trial in 36 women with adenomyosis, a significant decrease in abnormal uterine bleeding at the end of 3 months of 10 mg daily UPA was described. Unfortunately, there was no short-term investigation about the time to bleeding control (Capmas et al., 2021)

A Canadian retrospective, single-centre observational study of 163 premenopausal women with adenomyosis and/or fibroids examined a 12- week treatment of UPA. Amenorrhoea occurred in 90% of those with both adenomyosis and fibroids compared with 78% in the fibroids-only group (p = 0.0017). Optimal bleeding control (defined as PBAC < 75) was 90% and 74%, respectively (p = 0.028), suggesting the presence of adenomyosis had a favourable prognostic effect (Gracia et al. 2018).

### UPA and an Etonogestrel implant

Zigler et al. (2018) published a randomised, double-blind, placebo-controlled trial with 104 women aged 18–45 years with an Etonogestrel implant and more than one bleeding episode in a 24-day period. Fifteen mg UPA was administered daily for 7 days. After treatment, women randomised to UPA reported a significant decrease in bleeding days as compared to the placebo group. The median number of bleeding days was 7 versus 12 in the placebo group (p = 0.002). Bleeding cessation before day 10 was 10% in the placebo group versus 34% in the UPA group. However, this difference did not reach statistical significance.

### UPA and 52mg-Levonorgestrel (LNG)-IUD

In a randomised, double-blind, placebo-controlled pilot study, Fava et al. investigated the effect of 5 days of 5 mg UPA on the LNG-IUD bleeding pattern in 15 women. After 90 days of follow-up, a trend towards fewer days until bleeding cessation and more bleeding-free days in the intervention group was observed, although without reaching statistical significance (Fava et al., 2020; Henkel and Goldthwaite, 2020).

**Table I:** Median time to amenorrhoea (or bleeding control) per study and per dose of Ulipristal Acetate in 3-month administration *except for Chen et al., 2017. ± percentage interpreted from figure because no description of the exact numbers in the text.

Reference (Inclusion: fibroids with PBAC > 100 *)	Type of Study	UPA dose	Amenorrhoea		Definition amenorrhoea	Amenorrhoea AND/OR spotting < 10 d	Bleeding control (PBAC < 75)
			Median time	< 10 d			
Donnez et al., 2012a	Phase 3 RCT (Placebo-controlled)	5 mg		ca. 50%	PBAC ≤ 2 for 28 days		75.9% < day 7 ± 76% < day 10
		10 mg		ca. 70%			82.7% day 7 ± 84% day 10
Donnez et al., 2012b	non-inferiority RCT	5 mg	7d	± 57%	PBAC ≤ 2 for 28 days		± 86% < day 10
		10 mg	5d	± 80%			± 93% < day 10
Donnez et al., 2014	open label RCT	10 mg 1^st^ course	3.5 d [IQR]: 2-6	± 65%	35 days no bleeding (Max 1 day spotting)	± 81%	
		10 mg 2^nd^ course	2 d	± 86%		± 92%	
		10 mg 3^rd^ course	3 d	± 80%		± 87%	
		10 mg 4^th^ course	3 d	± 86%		± 92%	
Donnez et al., 2016	RCT	5 mg 1^st^ course	5 d (2-9)		35 days no bleeding (Max 1 day spotting)		
		10 mg 1^st^ course	4 d (2-7)				
Chen et al., 2017	Observational	5 mg		64%	Stop of bleeding		

## Discussion

Based on (1) UPA’s effectiveness in bleeding control in women with uterine fibroids, (2) the vast experience of single dose UPA in emergency contraception and (3) the knowledge of UPA’s pharmacokinetics, UPA may be considered in the early management of acute heavy uterine bleeding without fibroids.

The rapid clinical effect is due to its favourable pharmacokinetics, with a time to maximum concentration after intake of about 1 hour. The steepness of the incidence curve of bleeding control while administering it in patients with fibroids, suggests that the mechanism for short term bleeding control is a result of the effect of UPA on the endometrium and on the hypothalamic-pituitary- ovarian axis. The UPA induced inhibition of the myometrial collagen synthesis and the subsequent volume reduction of the myoma is slow and is not deemed to contribute to early bleeding control.

Long-term UPA intake may lead to benign, non-physiological endometrial changes, termed progesterone receptor modulator associated endometrial changes (PAEC) (Donnez et al., 2015; Singh and Belland, 2015). These changes tend to disappear completely within 6 months after ceasing the treatment (Donnez et al., 2015). In chronic UPA use, the development of endometrial hyperplasia or atypical hyperplasia has been reported in 0.7 and 0.2% respectively (Donnez et al., 2015). Long-term intake of UPA has also associated with hepatotoxicity (Singh and Belland, 2015). However, similar effects are not expected to occur after a short term, single dose exposure of UPA. A single 30 mg dose UPA is generally well tolerated. However, because of the possible toxicity after long term intake, repeating the single 30mg dosage should be considered with caution.

Ulipristal acetate may prove to be an alternative for the current standard of medical therapy in acute, heavy menstrual bleeding, being high dose (peroral or intravenous) oestrogens or gestogens. Oestrogens improve endometrial stability and growth while increasing the progesterone receptivity. High dose oestrogens are efficient in acute bleeding control but are associated with potential severe side-effects including deep venous thrombosis (ACOG committee opinion 2019). Hypertension, migraine with aura, history of deep venous thrombosis, overweight, smoking above 35 years, a history of breast cancer and liver disease are contraindications for oestrogen therapy.

Chronic progesterone administration converses the proliferative to secretory endometrium and eventually creates an atrophic endometrial lining (Bradley and Gueye, 2016). Continuous progesterone causes low serum levels of ovarian steroids, resulting in endometrial pseudo-decidualisation. The ability to decidualise the endometrium determines the progesterone’s efficacy in decreasing endometrial bleeding (Jewson et al., 2020). Progesterone is associated with weight gain, fatigue, depression, and spotting.

## Conclusion

UPA is increasingly used off-label for rapid bleeding control in premenopausal women without fibroids with acute heavy uterine bleeding. However, the review of the literature yielded only one case report on the use of UPA in the management of acute uterine bleeding unrelated to fibroids. Large, randomised studies in women with symptomatic fibroids already showed that the effect of UPA on bleeding control is rapid, effective, and probably independent of the presence or the volume of fibroids (Donnez et al., 2012a; Donnez et al., 2012b; Donnez et al., 2014; Donnez et al., 2015; Donnez et al., 2016). The effect tended to be better using 10 mg daily doses versus 5 mg. Serious side effects of UPA, in particular hepatotoxicity, have only been reported after longer use.

The vast clinical experience using UPA in emergency contraception has demonstrated the safety of a single 30 mg dose of UPA. Based on the drug’s pharmacokinetics, a single dose of 30 mg, if clinically indicated, repeated once on the fourth day, is expected to be at least as efficient as a one-week daily intake of 10 mg.

The vast clinical experience using UPA in emergency contraception has demonstrated the safety of a single 30 mg dose of UPA. Based on the drug’s pharmacokinetics, a single dose of 30 mg, if clinically indicated, repeated once on the fourth day, is expected to be at least as efficient as a one-week daily intake of 10 mg.

Whilst this is positive, the efficacy of a short course of UPA in the early management of heavy uterine bleeding should be validated in a large prospective study.
